# Prolonged air leak following lung resection - does Tri-staplerTM technology improve the incidence?

**DOI:** 10.1186/1749-8090-10-S1-A360

**Published:** 2015-12-16

**Authors:** V Srivastava, M Mydin, T Smailes, J Cotts, J Dunning

**Affiliations:** 1 Dept of Cardiothoracic Surgery, James Cook University Hospital, Middlesbrough, UK

## Background/Introduction

Prolonged air leak following lung resection leads to delayed discharge and increases risk of infection. The incidence of prolonged air leak (defined as greater than 7 days) is approx. 9% in the U.K. (SCTS Cardiothoracic Surgery database 2011). The Covidien Tri-staplerTM technology (Covidien, Mansfield, MA) claims to improve air leak rates following lung resection through improved vascularity at the suture line. We started using these staplers in August 2012.

## Aims/Objectives

To determine if Covidien Tri-staplersTM improve prolonged air leak incidence through comparison with the incidence in previous two years (i.e. August 2010 - July 2012).

## Method

The departmental database which collects and validates data prospectively was used to find patients having non-pneumothorax lung resection surgery between August 2010 and July 2014. They were divided into two groups - Group 1 (EndoGIA Autosuture™; August 2010 - July 2012) and Group 2 (Tri-stapler™ device; August 2012 - August 2014). The groups were then compared for preoperative variables and postoperative outcomes.

## Results

A total of 401 patients were included - Group 1 with 242 patients (102 males - 42.1%) and Group 2 with 159 patients (72 males - 45.3%). Mean age was 67.5 years (Group1) and 67.6 years (Group2); p = 0.92. COPD incidence was 59 (24.4%) patients in Group 1 and 66 (41.5%) patients in Group 2; p < 0.001. There was no significant differences in the incidence of prolonged air leak in Group 1 (n = 20; 8,3%) and Group 2 (13; 8.2%); p = 0.98. Significant infection prolonging hospital stay was more frequent in Group 1 (n = 17; 7%) than Group 2 (n = 18; 11.3%) but this was not statistically significant (p = 0.15). Mean post-operative stay was similar in both groups (7.9 days for Group 1 and 7.1 days in Group 2; p = 0.30).

## Discussion/Conclusion

The outcomes for the two groups were similar with no significant advantage from usage of the Tri-stapler™ technology.

